# Are Older Gamers Loners? Findings From a Country-Wide Study in the United Kingdom

**DOI:** 10.1093/geroni/igaf122.367

**Published:** 2025-12-31

**Authors:** Dwight Tse, Emma Anderson

**Affiliations:** University of Strathclyde, Glasgow, Scotland, United Kingdom; University of Strathclyde, Glasgow, Scotland, United Kingdom

## Abstract

The stereotypical perceptions of digital gamers as young and lonely are common. Do these perceptions apply to older gamers? In this presentation, we report findings from the UK Biobank data collected between 2006 and 2010, involving N > 70,000 participants aged 50 or older, who reported their computer gaming habits as “never” (non-gamers; 83%), “sometimes” (occasional gamers; 15%), or “often” (frequent gamers; 3%). Despite statistical significance driven by the large sample size, the effect sizes of differences in social engagement between gamers and non-gamers were mostly negligible to small, as indicated by measures of loneliness, family and friend visits, the ability to confide, and participation in leisure and social activities. Further exploratory results suggested negligible differences between older gamers and non-gamers in demographics (e.g., age, sex), sleep patterns (e.g., chronotype, insomnia), and psychological well-being (e.g., satisfaction with life, depressed mood). However, older gamers were less likely to adopt an active lifestyle and exhibited higher BMI, increased body fat, and lower self-rated physical health compared to non-gamers. These findings shed light on the distinct characteristics of older gamers and offer valuable perspectives for gerontologists and gaming industry professionals.

